# Supplementary material for: Identification of photoperiod-induced specific miRNAs in the adrenal glands of Sunite sheep (Ovis aries)
Source: Front Vet Sci. 2022 Jul 22;9:888207. doi: 10.3389/fvets.2022.888207 (PMC9354845; doi:10.3389/fvets.2022.888207)
Supplement: Supplementary file 1 [file Data_Sheet_1.ZIP › Supplementary file/miRDeep2 file.zip!/miRDeep2 file/Novel_Structure/SP42A3/Novel_347.pdf]

Provisional ID	:	NC_019473.2_23144
Score total	:	1.6
Score for star read(s)	:	-1.3
Score for read counts	:	0
Score for mfe	:	1.3
Score for randfold	:	1.6
Score for cons. seed	:	
Total read count	:	23
Mature read count	:	23
Loop read count	:	0
Star read count	:	0

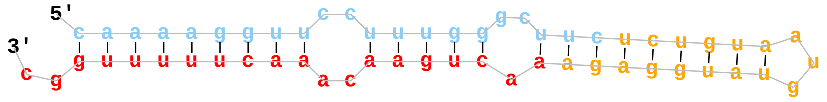

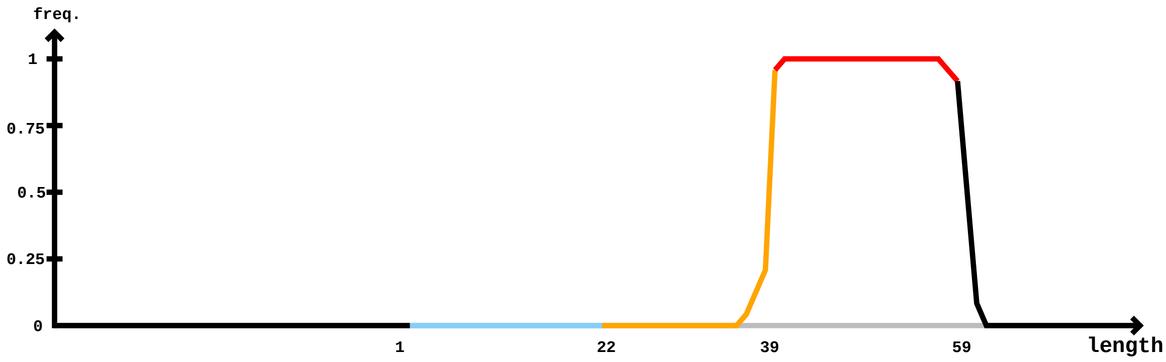


```
                                                   Star                                 Mature
5'- aauuaagucauagaauucagaaguacuggguuggccaaaagguuccuuugggcuucucuguaauguauggagaaacugaacaaacuuuuuggcaaaaucccauacgugcuu   -3'    exp
    ....................((((((((((...(((((((((((..(((((..(((((((((...))))))))).)))))..))))))))))).....))))...))))))    reads      mm        sample
    ......................................................................agaaacugaacaaacuuuuug....................    1          0         seq
    ........................................................................Aaaacugaacaaacuuuuugg..................    1          1         seq
    ........................................................................Aaaacugaacaaacuuuuuggc.................    1          1         seq
    .........................................................................aaUcugaacaaacuuuuuggca................    2          1         seq
    ..........................................................................aUcugaacaaacuuuuuggc.................    9          1         seq
    ..........................................................................aUcugaacaaacuuuuuggca................    8          1         seq
    ..........................................................................aUcugaacaaacuuuuuggcaa...............    1          1         seq
    ...........................................................................Ucugaacaaacuuuuuggcaa...............    1          1         seq
```
